# EZH2: a novel target for cancer treatment

**DOI:** 10.1186/s13045-020-00937-8

**Published:** 2020-07-28

**Authors:** Ran Duan, Wenfang Du, Weijian Guo

**Affiliations:** 1 Department of Medical Oncology, Fudan University Shanghai Cancer Center, Shanghai, 200032 People’s Republic of China; 2 Department of Oncology, Shanghai Medical College, Fudan University, Shanghai, 200032 People’s Republic of China; 3 Shanghai Medical College, Fudan University, Shanghai, 200032 People’s Republic of China

**Keywords:** EZH2, H3K27me3, Cancer, EZH2 inhibitor

## Abstract

Enhancer of zeste homolog 2 (EZH2) is enzymatic catalytic subunit of polycomb repressive complex 2 (PRC2) that can alter downstream target genes expression by trimethylation of Lys-27 in histone 3 (H3K27me3). EZH2 could also regulate gene expression in ways besides H3K27me3. Functions of EZH2 in cells proliferation, apoptosis, and senescence have been identified. Its important roles in the pathophysiology of cancer are now widely concerned. Therefore, targeting EZH2 for cancer therapy is a hot research topic now and different types of EZH2 inhibitors have been developed. In this review, we summarize the structure and action modes of EZH2, focusing on up-to-date findings regarding the role of EZH2 in cancer initiation, progression, metastasis, metabolism, drug resistance, and immunity regulation. Furtherly, we highlight the advance of targeting EZH2 therapies in experiments and clinical studies.

## Background

Transcription is important in cell fate decision. Dysregulation of transcription is a determinant of cancer phenotype. In addition to genetic alteration, abnormal epigenetic regulation of transcription plays an important role in carcinogenesis and cancer development.

Enhancer of zeste homolog 2 (EZH2) is a member of the family of polycomb group genes (PcGs), which is a group of important epigenetic regulators that repress transcription. Polycomb repressive complex 2 (PRC2) is one of the two PcG proteins core complexes and mediates gene silencing mostly by modulating chromatin structure [1]. EZH2 is an enzymatic catalytic subunit of PRC2 that can alter gene expression by trimethylation of Lys-27 in histone 3 (H3K27me3) [2]. H3K27me3 is associated with repression of genes expression and regarded as a critical epigenetic event during tissue development and stem cell fate determination [3]. Besides H3K27me3, PRC2 also methylates non-histone protein substrates, including the transcription factor GATA4 [4]. In addition, EZH2 methylates non-histone targets or directly interacts with other proteins to activate downstream genes in a PRC2-independent manner [5–7].

**Fig. 1:**
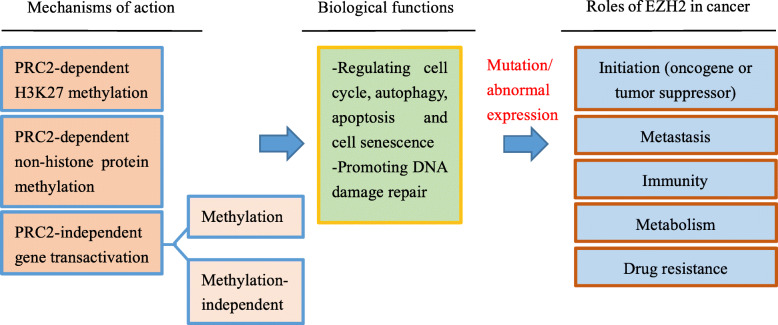
Mechanisms of action, functions, and abnormal changes of EZH2. EZH2 functions in various biological processes via 3 types of mechanism, including PRC2-dependent H3K27 methylation, PRC2-dependent non-histone protein methylation, and PRC2-independent gene transactivation. When mutation or abnormal expression happens, EZH2 is related to cancer initiation, metastasis, immunity, metabolism, and drug resistance

Via the above 3 ways, EZH2 works as a master regulator of cell cycle progression [8], autophagy, and apoptosis [9], promotes DNA damage repair and inhibits cellular senescence [10] and plays an important role in cell lineage determination and relative signaling pathways [11]. EZH2 functions in such various biological processes, which is the reason why it is related to many diseases, including cancer. The associations between EZH2 and cancer initiation, progression, metastasis, metabolism, drug resistance, and immunity regulation are key points discussed in this review (Fig. 1).

As there are many important roles of EZH2 in cancer, therapies targeting EZH2 have been important strategies in treatment of many types of cancer. This review discussed in detail about inhibitors of EZH2 methyltransferase activity, inhibitors that break PRC2’s structure, suppressing EZH2 through triggering EZH2 degradation, and the combination of EZH2 inhibitors with other treatment methods.

## Structure of EZH2

EZH2 gene is mapped to chromosome 7q35, including 20 exons coding 746 amino acid residues [12]. There are five domains, including EED-interaction domain (EID), Domain I, Domain II, cysteine-rich domain (CXC domain), and C-terminal suppressor of variegation 39, enhancer of zeste and trithorax domain (SET domain) [2, 13]. The histone methyltransferase activity of EZH2 is mainly maintained by SET domain, CXC domain N-terminal to the SET domain is also required. The N-terminal domains are the protein interaction domains, which are required for the assembly of partner subunits of proper PRC2 functions [2].

## Action modes of EZH2

EZH2 acts as a histone methyltransferase mainly by its SET domain, and it could suppress or co-activate transcription in a PRC2 dependent or independent way.

### PRC2-dependent H3K27 methylation

Histone, located in the center of nucleosome, is not just a packing protein, but a dynamic functional interface between DNA and other cell components. After histone modification, the chromosome structure changes. The enzyme that catalyzes histone modification in cells can transfer the information it carries to the chromosomal regulator, which eventually leads to the change of gene expression [14].

As a subunit of PRC2, EZH2 trimethylate H3K27 in the nucleus. PRC1 binds to PRC2-modified H3K27me3 and monoubiquitinylates histone H2A at lysine 119, which could facilitate chromatin compaction, and therefore promote downstream genes transcriptional silencing [15]. P21 is a key tumor suppressor gene, which inhibits cell proliferation by suppressing CDK. EZH2 could bind to P21 promoter region and mediate H3K27me3 modification, resulting in transcriptional silencing of P21, and promoting proliferation of gastric cancer cells [16].

### PRC2-dependent non-histone protein methylation

Besides histone, EZH2 also methylate non-histone proteins in a PRC2-dependent way. For example, EZH2 could methylate cardiac transcription factor GATA4 at Lys299 to decrease p300-mediated GATA4 acetylation and represses GATA4 transcription [4].

### PRC2-independent gene transactivation

EZH2 could activate downstream genes in a PRC2-independent way by direct methylation of non-histone proteins. AKT phosphorylates EZH2 at Ser21. STAT is a transcriptional factor, the phosphorylated EZH2 could methylate STAT3 to activate STAT3 signaling [5]. This PRC2-independent action mode was first found in castration-resistant prostate cancer (CRPC). In CRPC cells, phosphorylated EZH2 activates AR, which is a transcription factor, through a PRC2-independent but methylation-dependent mechanism. This activation of AR promotes transcription of its downstream genes and supports CRPC growth [6].

However, the most recent study revealed that EZH2 could play a non-catalytic role in CRPC cells in a PRC2- and methylation-independent way. EZH2 could directly activate AR gene transcription through occupying its promoter, which could not be blocked by enzymatic EZH2 inhibitor [7].

## Roles of EZH2 in cancer

Many members of PcG family play important roles in cancer, including Bmi-1 [17], Mel-18 [18], CBX7 [19], and EZH2. As previously described, EZH2 regulates its downstream genes and proteins by PRC2-dependent or PRC2-independent methylation or in a PRC2- and methylation-independent way, which are the basic mechanisms it works and shows so many functions mentioned before. However, when mutation happens or expression is abnormal, including overexpression, downregulated expression, and expression loss, it is related to cancer initiation, progression, and metastasis.

### Oncogenic role of EZH2

As EZH2 regulates cell cycle progression, dysregulation of EZH2 accelerates cell proliferation, and prolongs cell survival, which might lead to carcinogenesis and cancer development. Ectopic expression of EZH2 in breast epithelial cells could induce anchorage-independent colony growth and promote cell invasion, which is a demonstration of the oncogenic role of EZH2 [20]. EZH2 could inhibit apoptosis by regulation of E2F1-dependent apoptosis through epigenetically modulating Bim expression [21]. Different dysregulations are found in different types of cancers.

EZH2 overexpression was first identified in prostate cancer and associated with poor clinical outcomes [22]. More researches show evidences of EZH2 overexpression in many other cancer types, including breast cancer [23], esophageal cancer [24], gastric cancer [25], anaplastic thyroid carcinoma [26], nasopharyngeal carcinoma [27], and endometrial carcinoma [28]. IHC assessment of 62 samples shows that EZH2 expression is significantly higher in BAP1-mutant renal clear cell carcinoma patients with progressed stage, grade, nodal invasion, and metastasis [29]. Realtime qRT-PCR in 156 gastric cancer cases and immunoblot in randomly selected 20 cases showed that EZH2 was overexpressed in gastric cancer tissues compared with the matched adjacent normal mucous, and EZH2 overexpression is positively correlated with the tumor size, lymphatic invasion and TNM stage, and poor disease-free survival and overall survival of patients [25].

Different from the above solid tumor types, somatic mutations of EZH2 are identified in hematologic malignancies [30]. In B cell lymphoma, heterozygous point mutation occurred at the tyrosine 641 within the C-terminal catalytic SET domain of EZH2 [31]. This mutation mediates the gain-of-function of EZH2 enzymatic activity, therefore, promotes H3K27me3 and suppress gene expressions more strongly in lymphoma [32]. A meta-analysis of 8 studies including 2243 patients that evaluates the correlation between EZH2 mutations and overall survival in patients with myeloid neoplasms shows that EZH2 mutations were associated with significantly worse overall survival (hazard ratio = 2.37, 95% confidential interval, 1.48-3.79) [33].

### Role of EZH2 in cancer cells metastasis

A knockdown of EZH2 experiment indicates its potential role in metastasis. Human lung cancer cell lines-A549 and A129L cells were transfected with shRNA, migration ability was determined by wound healing assay and invasion ability was determined by transwell assay, and the results showed that knockdown of EZH2 markedly decreased migration and invasion ability of A549 and A129L cells. Besides, matrix-metalloproteinases (MMPs), which were closely related to the cancer cells invasion and metastasis, were downregulated by EZH2 knockdown [34].

An in vivo experiment proves the important role of EZH2 in cancer cells metastasis. In control melanoma model mice, melanoma-positive lymph nodes and distant metastasis in the lung are consistently developed; while in EZH2 conditional knockout mice, there is a significant reduction of lymph node metastasis and a virtual absence of lung metastasis [35].

Epithelial-mesenchymal transition (EMT) is the necessary and initial process of cell invasion and metastasis. In a pancreatic cancer cells experiment, exogenous overexpression of EZH2 resulted in increased expression of the mesenchymal marker Vimentin and decreased epithelial marker E-cadherin level, while EZH2 knockdown resulted in decreased expression of Vimentin and increased expression of E-cadherin [36]. These results reflected that EZH2 could promote EMT in pancreatic cancer cells.

Tumor angiogenesis plays an important role in tumor metastasis, EZH2 is also a key regulator of this process. The increase in endothelial EZH2 is a direct result of VEGF stimulation by a paracrine circuit that promotes angiogenesis by methylating and silencing vasohibin1 [37].

### Tumor suppressive roles of EZH2

While many researches have revealed oncogenic roles of EZH2, the tumor-suppressive roles are also reported. Cerulein could induce pancreatic injury. Histological changes include disordered acinar structure and abundant presence of metaplastic lesions. EZH2 is upregulated in the injured pancreatic tissue. Increased expression of EZH2 represses the CDK inhibitor *p16*^*Ink4a*^, therefore, controls the proliferative potential of PDX1-positive progenitor cells that accumulate transiently in metaplastic lesions, which is required during pancreatic repair. While loss of EZH2 blocks progenitor cells proliferation and redifferentiation into acini, thereby bringing regeneration to a halt and resulting in chronic injury. As chronic pancreatic injury is associated with high neoplastic risk, loss of EZH2 promotes neoplastic progression, which is also demonstrated by the mice experiment that loss of EHZ2 accelerates KRas^G12D^-driven neoplasia [38]. T cell acute lymphoblastic leukemia (T-ALL) is an immature hematopoietic malignancy driven mainly by oncogenic activation of NOTCH1 signaling, which induces loss of the repressive mark H3K27me3 by antagonizing the activity of PRC2 [39]. In Kras-driven lung adenocarcinoma, deletion and mutations of EZH2 are frequently present, and loss-of-function alterations are observed, which demonstrated that EZH2 might act as a tumor suppressor. Mechanistically, EZH2 loss amplified Akt and ERK activation through de-repressing its target insulin-like growth factor 1 [40]. An in vivo study shows that EZH2 concurrent knockout in mice with JAK2-V617F transgene resulted in a synergistic effect— very high platelet and neutrophil counts, more advanced myelofibrosis, and reduced survival. Moreover, JAK2-V617F–expressing mice treated with an EZH2 inhibitor showed higher platelet counts, mimicking the genetic knockout. These observations indicate that loss of EZH2 in hematopoietic cells might contribute to the development of myeloproliferative neoplasm; therefore, EZH2 might play a tumor-suppressive role [41].

### Role of EZH2 in cancer immunity

In the immune microenvironment of cancer cells, there are many kinds of immune cells and cytokines that interact with cancer cells to form a complex regulatory network. EZH2 expression in both cancer cells and immune cells have effects on cancer immunity.

EZH2 expression in cancer cells could suppress cancer immunity. EZH2 expression in ovarian tumor represses the tumor production of Th1-type chemokines CXCL9 [42], which plays a key role in CD8+ T cell infiltration in tumors [43]. The levels of EZH2 in ovarian tumor negatively correlated with intratumoral CD8^+^ T cells [42]. Tumor-associated macrophages (TAMs) acquired M2 phenotype could produce cytokines, including IL-6, IL-10, and C-C motif chemokine ligand 20 (CCL20), to reduce anti-tumor immune response and support tumor growth. EZH2 inhibition by siEZH2 in glioblastoma multiforme (GBM) cells could decrease the expression of M2 markers (TGFβ1-2, STAB1, Ym1, Lyvel, Fizz1, CD206, CD163) and increases the expression of M1 markers (TNFα and iNOS) in co-culturing microglia, which indicated that EZH2 in GBM cells promotes microglia shifting toward M2 phenotype. Moreover, EZH2 inhibition in GBM cells accelerated phagocytic capacities of TAMs. The mechanism is that as iNOS is increased, the production of NO is also increased, which plays an important role in the microglia phagocytic capacity under inflammatory conditions [44].

EZH2 expression in effector T cells and dendritic cells (DCs) could promote anti-cancer immunity. EZH2 expression in naïve T cells promotes survival, proliferation, and function of effector T cells [45]. EZH2 plays a key role for the function of tumor-specific effector T cells, while tumor microenvironments reduce EZH2 expression of T cells via glucose restriction and in turn, mediates T cell dysfunction [46]. The main function of DCs is antigen presentation and activation of primary T cells. EZH2 is necessary in the activation of DCs in allergic rhinitis and mediates epigenetic modification in allergen immunotherapy [47]. However, the effect of EZH2 on DCs in tumor microenvironment still needs further investigation.

Meanwhile, EZH2 expression in regulatory T cells (Tregs), helper T (Th) cells, and natural killer (NK) cells could suppress anti-cancer immunity. Tregs are critical for maintaining immune homeostasis as it suppresses immunity, but their presence in tumor tissues impairs anti-tumor immunity, thereby promoting tumor development and metastasis. Disruption of EZH2 activity in Tregs, either pharmacologically or genetically, drove the acquisition of pro-inflammatory functions in tumor-infiltrating Tregs, remodeling the tumor microenvironment and enhancing the recruitment and function of CD8+ and CD4+ effector T cells that eliminate tumors [48]. Th cells are important in the process of immune response and it could differentiate into many types of Th cells that play different roles. Inactivation of EZH2 could enhance Th1 and Th2 cell differentiation and plasticity [49]. NK cells participate in innate immune response, which can recognize and perform cytotoxicity against cancer cells. In the tumor microenvironment, NK cells differentiation and functions are repressed. Selective loss of EZH2 or inhibition of its enzymatic activity with small molecules in NK cells not only enhances NK cells commitment but also improves mature NK cells function, associated with upregulation of CD122 and the C-type lectin receptor NKG2D [50].

### Role of EZH2 in metabolism

Even when oxygen supply is enough, cancer cells obtain oxygen mainly from glycolysis, which is called the Warburg effect. In a prostate cancer study, the depletion of EZH2 inhibited prostate cancer cell growth and aerobic glycolysis accompanying the upregulation of miR-181b. EZH2 regulated aerobic glycolysis through miR-181b/hexokinase 2 (HK2) axis and played a positive role in cancer development [51].

EZH2 regulates adipocyte lipid metabolism. EZH2 inhibition promotes lipoprotein-dependent lipid accumulation via inducing ApoE expression in adipocytes [52]. In glioblastoma, siRNA-mediated EZH2 knockdown resulted in fatty acid synthase decrease, accompanied by diminished fatty acid levels. EZH2 regulates lipid metabolism through EZH2-TERT-lipid metabolism network [53].

### Role of EZH2 in drug resistance

Many cancer patients eventually generate resistance after treatment with chemotherapy drugs. Platinum drugs are used in first line to treat ovarian cancer, and most patients show high response to platinum drugs treatment initially, but nearly 80% of them develop recurrent tumors within 2 years and eventually generate acquired resistance against platinum drugs. Recent study revealed that EZH2 regulated cisplatin resistance in ovarian cancer through c-Myc-miR-137-EZH2 axis. Cisplatin treatment could induce production of ROS and generate cytotoxicity. Increased ROS production promoted expression of c-Myc, which activates expression of EZH2 by suppressing miR-137. High expression level of EZH2 could activate cell survival pathways, thereby promoting cisplatin resistance [54].

EZH2 is also associated with target drug resistance. Gefitinib is a tyrosine kinase inhibitor of epidermal growth factor receptor (EGFR), which is used to treat advanced EGFR mutated non-small-cell lung cancer (NSCLC). Recruited by long intergenic non-coding RNA 00665, EZH2 activated PI3K/AKT pathway and induced acquired resistance to gefitinib in NSCLC [55]. Tamoxifen, an antiestrogen drug, is used for the treatment of estrogen receptor (ER) positive breast cancer patients. EZH2 regulated tamoxifen resistance in breast cancer by the EZH2-ERα-GREB1 transcriptional axis [56].

## Targeting EZH2 drugs and strategy

As EZH2 plays important roles in the pathophysiology of cancer, it becomes a potential target for cancer therapy. Different types of EZH2 inhibitors have been developed, and there are a number of ongoing clinical trials of drugs targeting EZH2 in different cancer types (Table 1). The details can be inquired on the database of ClinicalTrials [57]. The classification and mechanisms of EZH2 inhibitors are shown in Table 2.

**Table 1:**
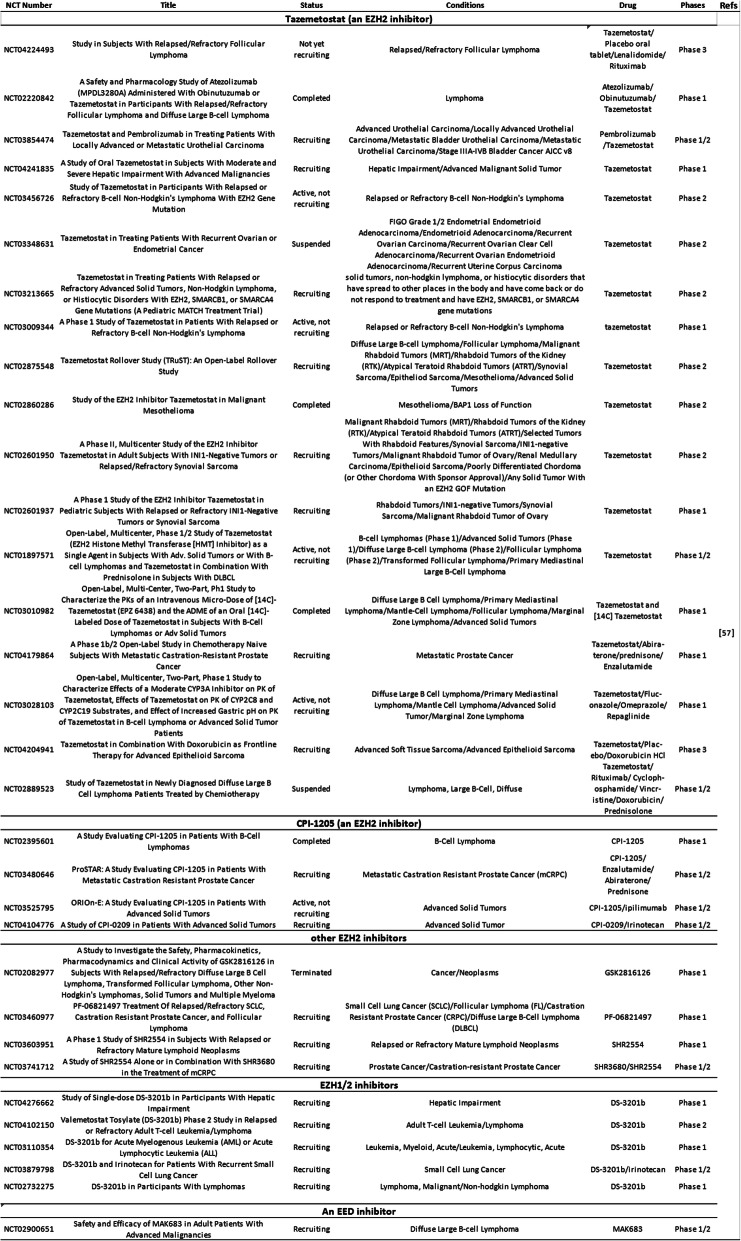
Clinical studies of drugs targeting EZH2

**Table 2:**
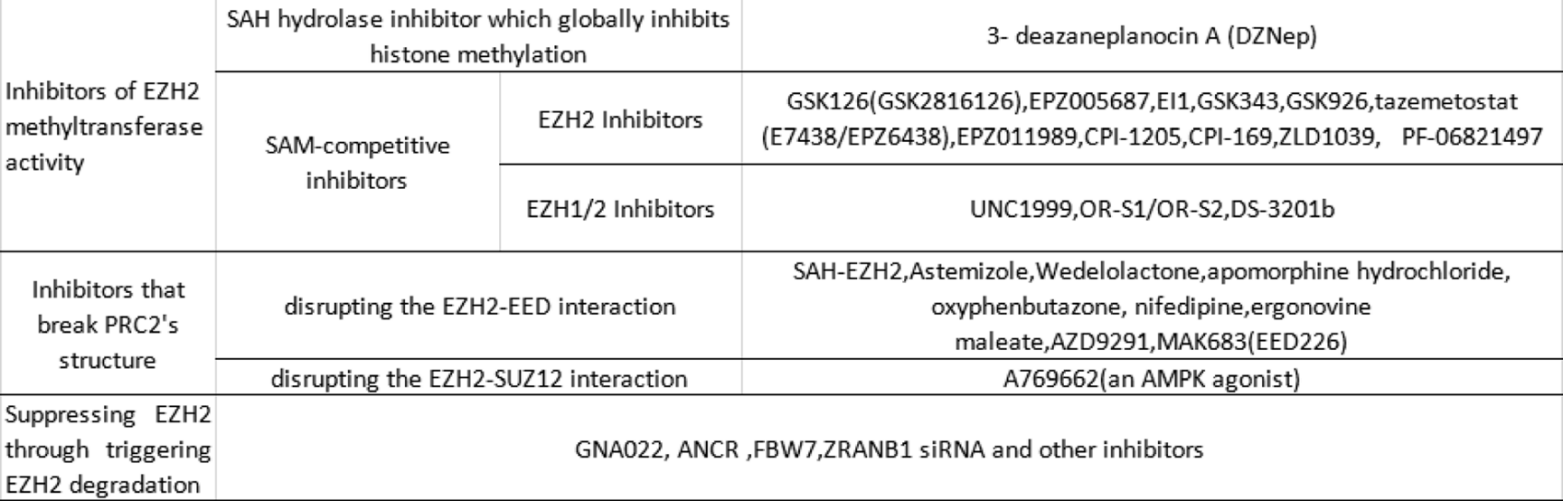
EZH2 inhibitors and their mechanisms of action

### Inhibitors of EZH2 methyltransferase activity

The first EZH2 inhibitor was 3-deazaneplanocin A (DZNep). DZNep, a known S-adenosyl-L-homocysteine (SAH) hydrolase inhibitor, indirectly inhibits EZH2 through the increase of SAH, which directly represses S-adenosyl-L-methionine-dependent histone methyltransferase activity. Thus, DZNep globally inhibits histone methylation and is not specific to EZH2 [58].

Since 2012, several potent and highly selective S-adenosyl-methionine-competitive inhibitors of EZH2 methyltransferase activity have been developed. Most of them have a common structural feature, a 2-pyridone core. The pyridone can occupy partially the site for the co-substrate S-adenosyl-methionine (SAM) in the binding pocket of EZH2 while SAM is the methyl donor [59].

GSK126 (GSK2816126) can inhibit wild type and Y641 mutant EZH2 with similar potency and is highly selective compared to EZH1 (150-fold increased potency) or 20 other methyltransferases (> 1000-fold selective for EZH2) [60]. A multicenter phase 1 clinical trial to evaluate the safety, maximum-tolerated dose (MTD), pharmacokinetics, and pharmacodynamics of GSK126 (NCT02082977) in patients with advanced hematologic and solid tumors was terminated. Forty-one participants (21 solid tumors, 20 lymphoma) received escalating doses of GSK2816126 ranged from 50 to 3000 mg twice weekly as an intravenous solution for 28 days (3 weeks on/1 week off) in the study. The results showed insufficient evidence of clinical activity, and did not justify further clinical investigation while the dosing method and relatively short half-life limited effective exposure [61]. In an experiment administering GSK126 treatment on tumor model mice, the results turned out that GSK126 had no effect on tumors in immunocompetent hosts, unlike that observed in immunodeficient hosts. The difference in the presence of myeloid-derived suppressor cells (MDSCs) between these two kinds of hosts might be the reason. MDSCs play an immunosuppressive role in the tumor microenvironment as it produces IL-6 and NO. GSK126 could promote hematopoietic progenitor cells differentiation to MDSCs, leading to increased MDSCs and immunosuppression, which might offset its antitumor effect on immune-competent hosts. Neutralizing antibodies against the myeloid differentiation antigen GR-1 or gemcitabine/5-fluorouracil can deplete MDSCs; thus, they can enhance the activity of GSK126 [62]. EPZ005687 [63], an EZH2 inhibitor concurrently developed with GSK126, with high affinity and selectivity for EZH2, has substandard pharmacokinetic properties that limit its clinical application. EI1 [64], another highly selective SAM-competitive inhibitor of EZH2, can inhibit the growth of DLBCL cells carrying Y641 mutations.

Later on, several other SAM-competitive inhibitors of EZH2 were developed including GSK343 [65], GSK926 [65], and tazemetostat (E7438/EPZ6438) [66]. GSK926 and GSK343 can suppress histone H3K27me3 level and inhibit EZH2 activity in breast and prostate cancer cells, while GSK343 can only be used in vitro due to the high clearance in rat PK studies [65]. Tazemetostat has improved potency and pharmacokinetic properties compared to EPZ005687 and can be taken orally in animals [67]. This compound is under evaluation in a series of clinical trials (Table 1). In a phase 2, multicenter, open-label, single arm, 2-stage study of tazemetostat 800 mg BID administered orally in continuous 28 day cycles (NCT02601950), patients were enrolled into seven cohorts based on tumor type after screening through 21 days of the first planned dose of tazemetostat [68]. In cohort 5, there were 62 patients (59 adults and 3 pediatric ≥ 16 years) with a documented diagnosis of metastatic or unresectable, locally advanced epithelioid sarcoma (ES). The objective response rate (ORR) for patients with ES was 15%, and the median duration of response (DOR) was 16 months. Sixty-eight percent of patients had a reduction in tumor burden. Median overall survival (mOS) was 19 months [69]. As for the clinical safety, tazemetostat shows a favorable safety and tolerability profile while most adverse events (AEs) are mild to moderate and resolved without the need for discontinuation or dose reductions [70]. Inspiringly, on January 23, 2020, tazemetostat (TAZVERIK, Epizyme, Inc.) was approved by FDA for adults and pediatric patients aged 16 years and older with metastatic or locally advanced epithelioid sarcoma not eligible for complete resection. Now, a phase 2, open-label, multi-center clinical trial for relapsed/refractory follicular lymphoma (FL) is ongoing [71]. As of the cutoff date, after screening and archival tissue analysis for EZH2 hot spot activating mutations, patients were enrolled into two cohorts (FL, EZH2 MT (cohort 1, *n* = 45) and FL, EZH2 WT (cohort 2, *n* = 54)). Patients were treated with tazemetostat 800 mg BID until progressive disease or withdrawal, and responses were assessed every 8 weeks. Treatment with tazemetostat was generally well tolerated and no treatment-related deaths were observed. The ORR for patients in cohort 1 was 77% and ORR in cohort 2 was 34%. Median DOR was 8.3 months in cohort 1 and 13 months in cohort 2. Median PFS was 11.1 months in cohort 1 and 5.7 months in cohort 2 (median DOR and PFS were not mature for the MT cohort). The results showed tazemetostat is a promising therapeutic drug for patients with relapsed/refractory follicular lymphoma.

EPZ011989 [72], another selective and orally bioavailable EZH2 inhibitor reported in 2015, was able to inhibit tumor growth significantly in a mouse xenograft model of human B cell lymphoma. Then, CPI-1205 [73], an orally bioavailable, indole-based, small-molecule inhibitor of EZH2 optimized from CPI-169 [74] was reported. CPI-169, a previously disclosed indole based EZH2 inhibitor, shows significant antitumor activity and pharmacodynamic (PD) target engagement in a mouse xenograft model of a KARPAS-422 lymphoma while accompanied by limited oral bioavailability [74]. CPI-1205 was evaluated in a completed phase 1 clinical trial for B cell lymphoma (NCT02395601). Furthermore, CPI-1205 is currently being evaluated in a phase 1/2 clinical trial for advanced solid tumors (NCT03525795) and a phase 1/2 clinical trial for metastatic castration-resistant prostate cancer (NCT03480646). ZLD1039 is a highly selective, and orally bioavailable inhibitor of EZH2, which inhibits breast tumor growth and metastasis in mice [75]. PF-06821497 [76] reported in 2018 is currently under evaluation in a phase 1 clinical trial in patients with relapsed/refractory small cell lung cancer (SCLC), castration-resistant prostate cancer (CRPC), FL and diffuse large B-cell lymphoma (DLBCL) (NCT03460977).

Given the fact that EZH1, a homolog of EZH2 physically presented in a non-canonical PRC2 complex, complements EZH2 in mediating H3K27 methylation and also has histone methyltransferase activity [77], dual EZH1/EZH2 inhibition may have greater antitumor efficacy. UNC1999 is the first oral SAM-competitive inhibitor of wild-type and Y641 mutant EZH2 as well as EZH1 [78]. UNC1999 effectively inhibited the growth of MLL-rearranged leukemia in mice instead of GSK126 in a study [79]. A more recent study introduced (R)-OR-S1 and (R)-OR-S2, two orally bioavailable EZH1/2 dual inhibitors produced by Daiichi Sankyo [80]. It was found that (R)-OR-S1 and (R)-OR-S2 suppressed H3K27me3 in HCT116 colorectal cancer cells more highly than OR-S0, an EZH2 selective inhibitor. Besides, (R)-OR-S1 and (R)-OR-S2 showed greater antitumor efficacy than OR-S0 in DLBCL cells harboring Y641N mutation of EZH2 both in vitro and in vivo. Despite of importance of EZH1 in hematopoietic stem cell maintenance [81], long-term EZH1/2 dual inhibition in vivo does not cause serious lympho-hematopoietic toxicity according to this study. Daiichi Sankyo soon put DS-3201b, an EZH1/2 inhibitor, into several clinical trials for patients with leukemia, lymphoma, or small cell lung cancer (NCT04276662, NCT03110354, NCT04102150, NCT02732275, NCT03879798).

### Inhibitors that break PRC2’s structure

In addition to targeting the enzyme catalytic domain of EZH2, disrupting the protein-protein interactions among the PRC2 subunits is a novel strategy to inhibit PRC2-dependent functions of EZH2.

Peptides known as stabilized alpha-helix of EZH2 (SAH-EZH2) were reported in 2013. SAH-EZH2, derived from the domain of EZH2 that interacts with EED, can disrupt the EZH2-EED interaction through targeting EED leading to an increased level of H3K27me3, reduced EZH2 protein and growth arrest, and differentiation of MLL-AF9 leukemic cells [82]. Furthermore, SAH-EZH2 impaired viability while GSK126 had no effect in MDA-MB231 (breast cancer) and DU145 (prostate cancer) cell lines which have been reported to be driven by non-enzymatic functions for EZH2 [82].

Then, several other inhibitors of the EZH2-EED interaction of PRC2 were identified. Astemizole, an FDA-approved H1 histamine receptor antagonist, was reported to arrest the proliferation of PRC2-driven lymphoma cells by disrupting the EZH2-EED complex [83]. Wedelolactone which has a high affinity for EED was screened out in natural compounds [84]. Four other FDA-approved drugs (apomorphine hydrochloride, oxyphenbutazone, nifedipine and ergonovine maleate) were discovered as potential EZH2-EED interaction inhibitors through a high-throughput fluorescence polarization assay [85]. A recent study found that AZD9291 (Osimertinib, TAGRISSO), a EGFR inhibitor approved by FDA for the treatment of patients with metastatic EGFR T790M mutation-positive NSCLC, can break the structure of EZH2-EED [85]. Besides, AZD9291 can also suppress the expression of EZH2 through upregulating miR-34a which can bind to EZH2 mRNA [86].

An EED inhibitor developed by Novartis is being evaluated in a phase 1/2 clinical trial for advanced malignancies including DLBCL, nasopharyngeal carcinoma, gastric cancer, ovarian cancer, prostate cancer, and sarcoma (NCT02900651). This inhibitor, MAK683/EED226, can make PRC2 allosteric through directly binding to the H3K27me3 pocket of EED [87].

Apart from targeting EZH2-EED interaction, disrupting the interaction between EZH2 and SUZ12 was reported in a study [88]. In this study, they demonstrated that AMP-activated protein kinase (AMPK) can disrupt the EZH2-SUZ12 interaction through directly phosphorylating EZH2 at Thr311 and decrease the level of H3K27me3 in ovarian and breast cancer cells [88].

### Suppressing EZH2 through triggering EZH2 degradation

Given the fact that EZH2 plays a PRC2- and methylation-independent role in cancer and many cancers do not respond to EZH2 enzymatic inhibitors, triggering EZH2 degradation may be a novel method to inhibit EZH2.

In addition to the abovementioned SAH-EZH2 reducing EZH2 protein through disrupting the PRC2 complex, post-translational modifications of EZH2 can aslo induce the degradation of EZH2. Wang et al. reported a gambogenic acid (GNA) derivative, GNA022, directly covalently bound to Cys668 within the EZH2-SET domain, decreasing the stability of PRC2 complex as well as H3K27 trimethylation, triggering EZH2 degradation through COOH terminus of Hsp70-interacting protein (CHIP)-mediated ubiquitination [89]. Another group found that long non-coding RNA (lncRNA) ANCR can facilitate the CDK1-EZH2 interaction, then phosphorylate EZH2 at Thr345 and Thr487, hence, result in EZH2 ubiquitination and its degradation in breast cancer cells in vitro, and ANCR represses tumor growth and distant metastasis in mice in breast cancer [90]. F-box and WD repeat domain-containing 7 (FBW7), a novel E3 ligase of EZH2, can mediate the phosphorylation, ubiquitination, and degradation of EZH2 with the involvement of the activated CDK5 kinase in pancreatic cancer [91].

Lu et al. revealed a new signaling network of SKP2-TRAF6-EZH2/ H3K27me3 and found knockout of SKP2 can upregulate TRAF6-mediated and lysine (K) 63-linked ubiquitination of EZH2 for degradation in prostate cancer (PCa) and CRPC cells in vitro and in vivo [92]. In a study in 2018, ZRANB1 was identified as the EZH2 deubiquitinase and stabilizes EZH2 through interacting with EZH2 via its OTU domain in breast cancer cells. Thus, ZRANB1 small interfering RNA (siRNA) and other ZRANB1 inhibitors have anticancer effects in vitro and in vivo [93].

### EZH2 inhibitors combined with other therapy methods

Combining EZH2 inhibitors with other therapy methods such as immune therapy, conventional chemotherapy, and target therapy might improve the treatment efficacy and overcome the limitation of monotherapy.

As mentioned before, the levels of EZH2 negatively correlated with intratumoral CD8+ T cells in ovarian cancer, so EZH2 inhibitor could increase effector T cell tumor infiltration, slowed down tumor progression, and synergistically improved the efficacy of adoptive T cell therapy [45]. Furthermore, EZH2 inhibition can increase the cytotoxicity of human effector T cells in vitro and improve the efficacy of anti-CTLA-4 therapy in murine bladder cancer and melanoma as anti-CTLA-4 increases the expression of EZH2 in peripheral T cells [94]. This study provided basis for the treatment of combining CPI-1205 with ipilimumab (anti-CTLA-4). In a phase 1/2, multi-center, open-label study (NCT03525795), the strategy of CPI-1205 plus ipilimumab is under evaluation in patients with advanced solid tumors. A recent study revealed the inhibition of EZH2 could enhance cancer cell antigen presentation in head and neck squamous cell carcinoma (HNSCC) and avoid anti-PD-1 resistance [95].

Synergy between EZH2 inhibitors and conventional chemotherapy has been demonstrated in a series of preclinical studies. Synergistic effects of tazemetostat with cyclophosphamide, doxorubicin, oncovin, and prednisone were observed in EZH2 mutant DLBCL [96]. In a multicenter, double-blind, placebo-controlled, randomized phase 3 clinical trial (NCT04204941), the combination of tazemetostat with doxorubicin for patients with advanced ES is being evaluated. Part 1 is designed to evaluate the safety of the combination of tazemetostat plus doxorubicin, as well as to establish the MTD and the recommended dose for part 2. Part 2 is planned to compare the effects of tazemetostat plus doxorubicin with doxorubicin plus placebo, when used as first-line treatment in locally advanced unresectable or metastatic ES [97]. Besides, tazemetostat plus R-CHOP combination (rituximab, cyclophosphamide, vincristine, doxorubicin, prednisolone) in patients with newly diagnosed DLBCL with poor prognosis features is under evaluation in a phase 1/2 clinical trial (NCT02889523). Phase 1 is designed to determine the recommended phase 2 dose for tazemetostat in patients treated with R-CHOP 21. Phase 2 is designed to determine the safety of tazemetostat in patients treated with 8 cycles of R-CHOP 21 and to determine the complete response rate after 8 cycles of Epi-RCHOP (tazemetostat plus RCHOP) 21. Up to present, 17 patients were enrolled and further evaluation in phase 2 is warranted while this therapeutic method showed preliminary efficacy in phase 1 [98].

Several studies demonstrated co-inhibition of both EZH2 and EGFR could show a synergic effect on tumor growth inhibition through inducing autophagy and increasing apoptosis in colon cancer cells [99], gastric cancer cells [100], and lung cancer cells [101]. Besides, this combination therapy can reverse EGFR-tyrosine kinase inhibitor (EGFR-TKIs) resistance in lung cancer [99]. Futhermore, Hirukawa et al. in 2019 found EZH2 inhibitors could enhance the efficacy of anti-HER2 monoclonal antibodies such as trastuzumab through promoting interferon-driven immune responses in trastuzumab-resistant breast cancer models of mice [102]. As previously mentioned, phosphorylated EZH2 activates AR and the activation of AR supports CRPC growth, so combination of EZH2 inhibition and AR-targeted therapies may be effective in CRPR [6]. There are two ongoing clinical trials to evaluate the effects of EZH2 inhibitors plus enzalutamide (an AR antagonist) in mCRPR (NCT04179864, NCT03480646).

To overcome the limitation that EZH2 inhibitors can only benefit certain hematological malignancies, Huang et al. in 2018 found BRD4 inhibitors can decrease the resistance of EZH2 inhibitors caused by H3K27ac upregulation and restore the sensitivity of the insensitive cell lines to EZH2 inhibitors [103]. Besides, as EZH2-BRD4 inhibitor combo differentially activates multiple pathways such as MAPK pathway, a triple combination plus MAPK pathway inhibitors may expand the treatment scope of cancers [103]. However, the therapeutic effects of multi-drug combination require further evaluation in clinical trials, and the side effects and patients’ tolerance may be a big problem.

## Conclusions

As a novel and potential target for cancer therapy, EZH2 has become a hotspot of research. More and more functions and roles of EZH2 in multiple kinds of cancer have been revealed. Many new drugs targeting EZH2 are under development and evaluation in clinical trials. Most of them are still on the stage of preclinical study or phase 1/2 clinical trials, only tazemetostat was approved for the treatment of ES and preliminarily showed promising effects on FL.

To further develop EZH2 inhibitors with high efficiency, low toxicity, and high selectivity is one of the future directions. Dual EZH1/EZH2 inhibition may have greater anti-tumor efficacy because EZH1 can compensate when EZH2 is inhibited. So dual EZH1/EZH2 inhibitors targeting their common sequence with high selectivity or combining highly selective EZH1 inhibitors with highly selective EZH2 inhibitors are both worth being explored. Besides, conducting more clinical trials to evaluate and verify the efficacy of EZH2 inhibitors is significant. Screening predictive biomarkers, such as EZH2 mutation or over-expression, to help select patients suitable for EZH2 target therapy is important in personalized, precision therapy. Biomarker-based selection of patients registered in the clinical trial will be a better strategy to improve the efficacy of EZH2 inhibitor and increase the chance of positive results. Given the fact that single drug has limited efficacy, combining EZH2 inhibitors with other treatments is an essential option and future direction, as pre-clinical studies have already shown that EZH2 inhibitors combined with immunotherapy or chemotherapy has a synergistic effect and has entered the stage of clinical trials.

## Data Availability

Data sharing is not applicable to this article as no datasets were generated or analyzed during the current study.

## Abbreviations

AEs: Adverse events
AMPK: AMP-activated protein kinase
AR: Androgen receptor
CCL20: C-C motif chemokine ligand 20
CHIP: COOH terminus of Hsp70-interacting protein
CRPC: Castration resistant prostate cancer
DCs: Dendritic cells
DLBCL: Diffuse large B cell lymphoma
DOR: Duration of response
DZNep: 3-deazaneplanocin A
EGFR: Epidermal growth factor receptor
EGFR-TKIs: EGFR-tyrosine kinase inhibitor
EMT: Epithelial-mesenchymal transition
ER: Estrogen receptor
ES: Epithelioid sarcoma
EZH2: Enhancer of zeste homolog 2
FBW7: F-box and WD repeat domain-containing 7
FL: Follicular lymphoma
GBM: Glioblastoma multiforme
GNA: Gambogenic acid
H3K27me3: Trimethylation of Lys-27 in histone 3
HK2: Hexokinase 2
HNSCC: Head and neck squamous cell carcinoma
lncRNA: Long non-coding RNA
MDSCs: Myeloid-derived suppressor cells
MMPs: Matrix-metalloproteinases
mOS: Median overall survival
MTD: Maximum-tolerated dose
NK: Natural killer
NSCLC: Non-small-cell lung cancer
ORR: Objective response rate
PCa: Prostate cancer
PcGs: Polycomb group genes
PD: Pharmacodynamics
PRC2: Polycomb repressive complex 2
R-CHOP: Rituximab, cyclophosphamide, vincristine, doxorubicin, prednisolone
SAH: S-adenosyl-L-homocysteine
SAH-EZH2: Stabilized alpha-helix of EZH2
SAM: S-adenosyl-methionine
SCLC: Small cell lung cancer
siRNA: Small interfering RNA
T-ALL: T cell acute lymphoblastic leukemia
TAMs: Tumor-associated macrophages
Th cells: Helper T cells
Tregs: Regulatory T cells
